# Supplementary material for: Mental toll on working women during the COVID-19 pandemic: An exploratory study using Reddit data
Source: PLoS One. 2023 Jan 17;18(1):e0280049. doi: 10.1371/journal.pone.0280049 (PMC9844921; doi:10.1371/journal.pone.0280049)
Supplement: Attachment — Submitted filename: Rebuttal Letter.docx [file pone.0280049.s002.docx]

Journal Requirements:

When submitting your revision, we need you to address these additional requirements.

1. Please ensure that your manuscript meets PLOS ONE's style requirements, including those for file naming. The PLOS ONE style templates can be found at
   <https://journals.plos.org/plosone/s/file?id=wjVg/PLOSOne_formatting_sample_main_body.pdf> and 
   <https://journals.plos.org/plosone/s/file?id=ba62/PLOSOne_formatting_sample_title_authors_affiliations.pdf>

*We have made the necessary changes.*

2. In your Methods section, please include additional information about your dataset and ensure that you have included a statement specifying whether the collection and analysis method complied with the terms and conditions for the source of the data.

*We have added the extra information as requested.*

3. In your Data Availability statement, you have not specified where the minimal data set underlying the results described in your manuscript can be found. PLOS defines a study's minimal data set as the underlying data used to reach the conclusions drawn in the manuscript and any additional data required to replicate the reported study findings in their entirety. All PLOS journals require that the minimal data set be made fully available. For more information about our data policy, please see <http://journals.plos.org/plosone/s/data-availability>.

"Upon re-submitting your revised manuscript, please upload your study’s minimal underlying data set as either Supporting Information files or to a stable, public repository and include the relevant URLs, DOIs, or accession numbers within your revised cover letter. For a list of acceptable repositories, please see <http://journals.plos.org/plosone/s/data-availability#loc-recommended-repositories>. Any potentially identifying patient information must be fully anonymized.

*Done.*

4. PLOS requires an ORCID iD for the corresponding author in Editorial Manager on papers submitted after December 6th, 2016. Please ensure that you have an ORCID iD and that it is validated in Editorial Manager. To do this, go to ‘Update my Information’ (in the upper left-hand corner of the main menu), and click on the Fetch/Validate link next to the ORCID field. This will take you to the ORCID site and allow you to create a new iD or authenticate a pre-existing iD in Editorial Manager. Please see the following video for instructions on linking an ORCID iD to your Editorial Manager account: <https://www.youtube.com/watch?v=_xcclfuvtxQ>

*Done.*

5. Please include a separate caption for each figure in your manuscript.

*Done.*

6. Please include captions for your Supporting Information files at the end of your manuscript, and update any in-text citations to match accordingly. Please see our Supporting Information guidelines for more information: <http://journals.plos.org/plosone/s/supporting-information>.

*Done.*

**Comments to the Author**

5. Review Comments to the Author

Please use the space provided to explain your answers to the questions above. You may also include additional comments for the author, including concerns about dual publication, research ethics, or publication ethics. (Please upload your review as an attachment if it exceeds 20,000 characters)

**Reviewer #1:**

This paper used social media data to test whether there were significant different in people’s mental health in terms of unemployment during the COVID-19 epidemics. Nature language processing and MANOVA were adopted to test the hypotheses, and the results supported the arguments. The language is fluent with few spelling or grammar errors. The research topic is quite interesting and important.

However, there are several issues that I would like to mention:

1. One major concern I have is that the research data and results may not be able to answer all the research questions. The aim of this research was “to identify if mental health issues were heightened due to age- and gender-based inequity in terms of unemployment during the pandemic”. While the results showed that “that younger women express their vulnerability on social media more strongly than older women or men.” Men have been proved to be less likely to express their vulnerability. And even they are talking about games on social media platform, they may actually be experiencing huge stress at the same time. The authors should justify whether they measured the expressed health issues or the actual health issues.

*We have added a clarifying statement noting that we have measured only the expressed health issues and not the actual health issues.*

2. One of the literature review part names “predicting user gender and age”. However, there is only one literature in that part was talking about predicting gender and age, other reviewed literatures were talking about the importance of related topic. More related literatures on “predicting user gender and age” should be reviewed or the authors could consider changing a sub-title.

*We have changed the sub-title to emphasize the relevance of gender and age.*

3. Sharp pictures with higher resolution should be provided.

*We have added pictures with higher resolution.*

**Reviewer #2:**

1. Pg. 2, background – helpful to provide the 1948 unemployment rate number as a reference

*We have added the reference.*

2. Pg. 5, Objective – after reading this objective and the background, it seems that the authors are summarizing findings from existing studies but not addressing a clear research gap. It would be helpful if the authors could elaborate on the literature gaps or weaknesses of existing works and how this work could potentially help.

*We have added a paragraph under the “objective” section clearly stating the research gap and how our work can potentially help.*

3. Pg. 5-6, data selection & comparison groups – is it a 1-to-1 match or propensity score matching? What are the three pairs? Those four groups seem naturally to be four pairs: 1) women under 30 vs. over 30; 2) men under 30 vs. over 30; 3) under 30 women vs. men; 4) over 30 women vs. men. Based on later sections, the authors seem to be using three dimensions (sentiment, stress, and loneliness), but they are still different from the definition of pairs. Elaborating on this could help remove confusion.

*We apologize for the confusion. We have clarified the difference between pairs and dimensions by explicitly stating the four pairs and three dimensions used in this study.*

4. Pg. 7, results – figures have low resolutions. For example, figure 1 (original graph added as supplemental material) has a horizontal and vertical DPI of 96, which is lower than the 300-dpi standard journal requirements. I would suggest the authors regenerate those in higher resolutions to be legible. Besides, figures 1 and 2 did not provide any legends.

*We have added pictures with higher resolution.*

5. Pg. 7, results – “We used a Bonferroni corrected alpha was used to account for the multiple ANOVA tests,” – this is a grammatically wrong sentence (used appeared twice with unclear subjective). Please double-check the manuscript for such errors.

*We apologize for this error. We have made the correction.*

6. Pg. 7, results – “We can also see from the descriptive statistics table (Table 3)” – there are no statistical significance levels reported in Table 3, so these interpretations may not be statistically significant.

*We do not claim that it is statistically significant.*

7. Pg. 9, principal results – “Unemployed and work from home redditors in 2020 are psychologically affected by the pandemic.” This statement is not supported by the results. To address this, the author could do a thematic analysis of texts mentioning “pandemic” in the reddits. A casual or association relationship could not be established because these reddits happened during the pandemic – concurrence does not mean causal relationships. Their scenarios could be heterogeneous and may not be uniformly influenced by the pandemic.

*Since this is not the main focus of the paper, we have removed the sentence in question.*

8. Pg. 10, limitations – another limitation is the digital world could not represent the entire world, both the coverage and depth of the messages. Still, a large group of people would not have regular access to the internet or prefer not to engage in social media discussions. The authors should mention the bias from the unobserved population. On the other hand, posts expressed on social media are edited versions of reality so that they could be exaggerated or distorted.

*We have added a limitation that talks about this.*

**Reviewer #3:**

1. In the "Data" section, the authors reported that they collected data from the unemployment subreddit and work from home subreddit. But in the next paragraph, the authors only reported about the time frame for collecting data from the unemployment subreddits. Please clarify the time frame and relevant details for collecting data from work from home subreddit.

*Apologies for the confusion. We have removed work from home users from the study and mention of it from the manuscript.*

2. In the "Data Selection" section, the authors mentioned that they sampled 1500 redditors in each of the four groups. Please add more details about how were these redditors sampled.

*We used 1:1 matching pair design to make sure each pair is matched on gender and age. In each group, there is equal representation of both gender and age groups. Data sampling was repeated three times to avoid selection bias. Finally, we had an unbiased sample of 1,500 redditors in each of the four focus groups: women under 30, women over 30, men under 30, and men over 30.*

3. In the "Well-being Measurements" section, the authors reported that they used NLP tools to assess sentiment, stress, and loneliness. How good are the validity of these tools? Please clarify if possible.

*There are several studies mentioned in the manuscript that talk about these tools and their performances.*

4. In general, the authors should be more explicit about their contribution to the existent literature.

*We have added a paragraph under the “objective” section clearly stating the research gap and how our work can potentially help.*
