# Supplementary material for: Mental toll on working women during the COVID-19 pandemic: An exploratory study using Reddit data
Source: PLoS One. 2023 Jan 17;18(1):e0280049. doi: 10.1371/journal.pone.0280049 (PMC9844921; doi:10.1371/journal.pone.0280049)
Supplement: Attachment — Submitted filename: Rebuttal Letter.docx [file pone.0280049.s003.docx]

**Rebuttal Letter**

**Journal Requirements:**

Please review your reference list to ensure that it is complete and correct. If you have cited papers that have been retracted, please include the rationale for doing so in the manuscript text, or remove these references and replace them with relevant current references. Any changes to the reference list should be mentioned in the rebuttal letter that accompanies your revised manuscript. If you need to cite a retracted article, indicate the article’s retracted status in the References list and also include a citation and full reference for the retraction notice.

*There was only one reference that was from a website that is longer present, so we removed it. A few of the references did not show the hyperlinks correctly or had some sort of problem due to formatting. We made changes to all of them and now all the references should be valid and easily accessible. Hope this helps with this issue raised.*

**Review Comments to the Author**

**Reviewer #1:** The authors have addressed majority questions raised by the Editor and reviewers. It would be more helpful if the authors could provide the location and brief summary for some of the modifications in the response letter in the future.

I am happy to recommend this manuscript for publication after more proof reading and format correction.

*Thank you for the suggestion. We will keep this in mind for the future.*

**Reviewer #2:**

1. Figures still need higher resolution and legends (maybe revised figures got lost in transmission?). I will defer to the journal editor and production team on how to address this.

*We tried our best to address this issue and hopefully we have solved the problem of resolution this time. We have double checked the resolution and the legends in the figures.*

2. Limitations: “Social media data also poses the threat of selection bias.” This one sentence may not be sufficient. Please make sure to elaborate a bit more on the “how”.

*We added the following to the limitation section:*

*“Social media data also poses the threat of selection bias. Regardless how widely used Reddit is, social media data might not represent the entire real-world population. As we mentioned earlier when discussing the data we used, Reddit has particular biases in its user population, especially in gender and age.”*

**Reviewer #3:** (No Response)
